# Microbial diversity in shallow-sea sediment from Tsukumo Bay, Japan, determined by 16S rRNA gene amplicon sequencing

**DOI:** 10.1128/mra.01061-23

**Published:** 2024-03-18

**Authors:** Moe Kato, Reina Sugitani, Sota Niiyama, Takahiro Kamiya, Norihisa Matsuura

**Affiliations:** 1 Faculty of Geosciences and Civil Engineering, Kanazawa University, Ishikawa, Japan; 2 School of Geosciences and Civil Engineering, Kanazawa University, Ishikawa, Japan; 3 Okinawa Prefectural Museum and Art Museum, Okinawa, Japan; Montana State University, Bozeman, Montana, USA

**Keywords:** marine sediment, sulfate reduction, 16S amplicon sequence

## Abstract

Information about the microbiota in marine sediments is important because the microbiota and their activities in sediments affect the surrounding marine environment. To evaluate the microbial diversity, we performed 16S rRNA gene amplicon sequencing on sediment samples from 19 stations in Tsukumo Bay, the northern area of Noto Peninsula, Japan.

## ANNOUNCEMENT

Marine sediments contain abundant microorganisms that play important roles in biogeochemical element cycling (1–3). The diversity and distribution patterns of marine microbiota in marine sediments are significantly affected by multiple environmental factors (4). Therefore, studying the microbiota is important to understand the interrelationship between microorganisms and marine environments. Tsukumo Bay (37.305°N, 137.236°E) is an inlet on the Noto Peninsula, Japan, 1,300 m long, 250 m wide, and 25 m deep in the central region. Tsukumo Bay is maintained in a stable environment inside the bay generally because the entrance to Tsukumo Bay is relatively narrow and shallow (5), and some types of bottom sediments are distributed there. In this study, 16S rRNA gene amplicon sequencing was used to explore the diversity of microbial communities in sediment samples collected from various locations in Tsukumo Bay, Japan.

**Fig 1:**
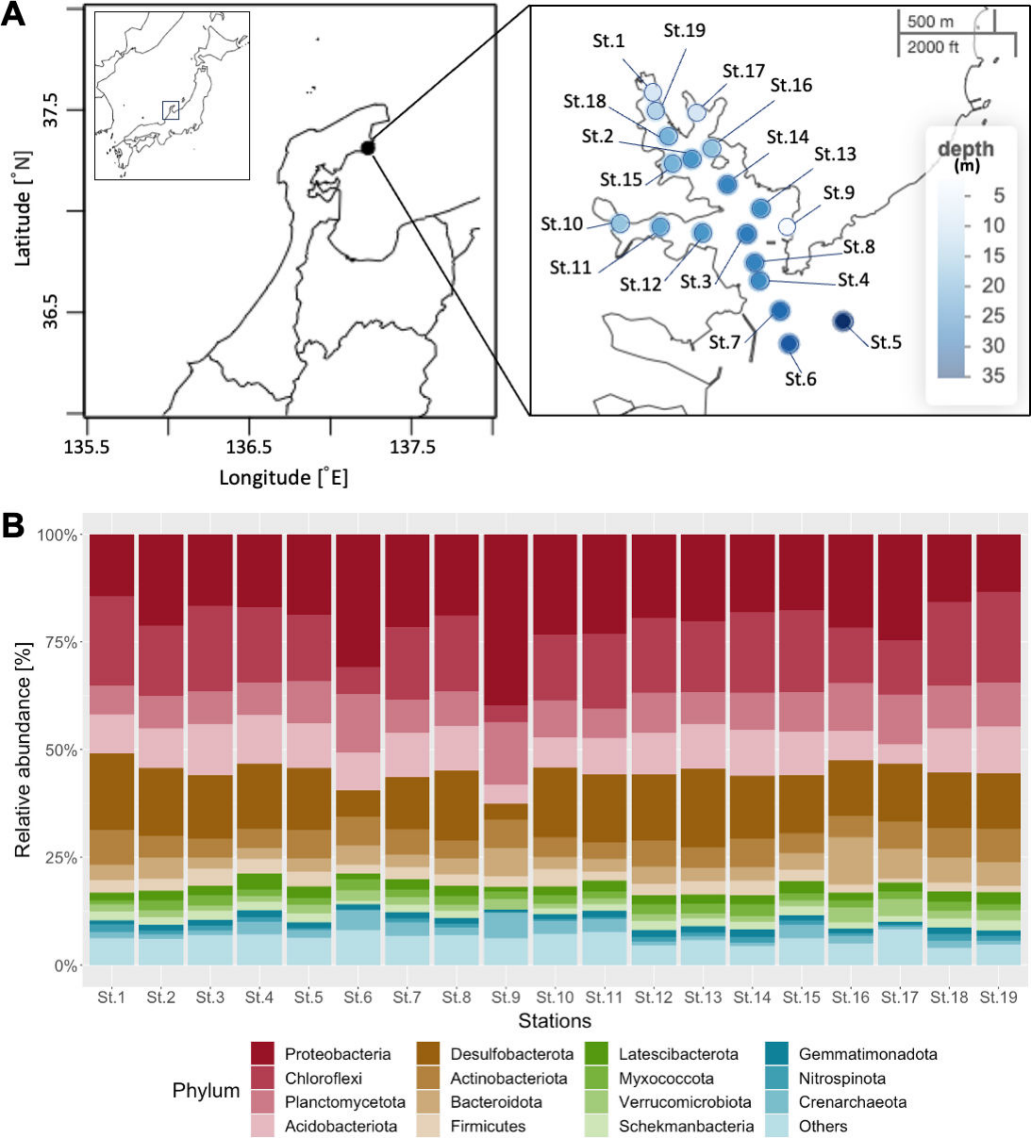
(A) Map of the sampling stations in Tsukumo Bay (Noto Peninsula, Ishikawa, Japan). Each circle color represents the water depth at each sampling station. The maps were obtained from GSI Maps published by the Geospatial Information Authority of Japan. (B) Column chart showing the relative abundance of the top 15 microbial phyla at each sampling station.

Sediment samples were collected as core samples using plastic cylinders (φ28 mm) from 19 stations (Stations 1–19) in Tsukumo Bay, the northern area of Noto Peninsula, Japan (Fig. 1; Table 1), in May 2022. The samples were stored in a cooler with ice packs for about 3 hours and transferred to the lab, where they were stored at −20°C. Surface sediments (0–2 cm in depth) were used for 16S rRNA gene amplicon sequencing. After homogenizing each sample by using a Vortex-Genie 2 (Scientific Industries, USA), total DNA was extracted using the DNeasy PowerSoil Pro kit and automatic DNA extractor QIAcube Connect (Qiagen, Germany), following the manufacturer’s protocol. Amplicon libraries were prepared using a two-step tailed PCR method (6) with 515F and 806R primers targeting the V4 region of the prokaryotic 16S rRNA gene (7). Illumina adapter sequences were attached to the 5′ end of the forward and reverse sequencing primers. The prepared libraries were sequenced on an Illumina MiSeq platform at Kanazawa University using the MiSeq Reagent Nano Kit v2 (Illumina, USA). PhiX Control v3 (Illumina) was added to the libraries at a concentration of approximately 30% (vol/vol). This yielded 208,870 reads for the 19 data sets of the 16S rRNA gene. The 2 × 250-bp paired-end reads were trimmed (options: TRUNCLENF = 200, TRUNCLENR = 170), quality-filtered, denoised, and merged. Amplicon sequence variant (ASV) inference was performed, chimeras were removed, and ASVs were generated using DADA2 v1.16.0 (8). Taxonomic assignments were performed using the SILVA 138 SSU Ref NR 99 database (9). The default settings were used for all software unless otherwise indicated.

**TABLE 1:** Summary of sediment samples and the sequencing results from Tsukumo Bay

Sample ID	Latitude	Longitude	Depth (m)	Sediment type	No. of raw sequencing reads	DRA accession no.
Station 1	37.31104999°N	137.2311828°E	7.3	Mud	8,817	DRR511521
Station 2	37.30804999°N	137.2334169°E	22.1	Mud	7,009	DRR511522
Station 3	37.30466694°N	137.2365669°E	25.5	Mud	9,569	DRR511523
Station 4	37.30254972°N	137.2372497°E	23.8	Sandy mud	4,964	DRR511524
Station 5	37.30068277°N	137.2420828°E	35.1	Sandy mud	10,429	DRR511525
Station 6	37.29966694°N	137.2389497°E	29.8	Sand	11,099	DRR511526
Station 7	37.30116694°N	137.23845°E	27.6	Sandy mud	15,430	DRR511527
Station 8	37.30336694°N	137.2370169°E	24.5	Sandy mud	8,463	DRR511528
Station 9	37.30491694°N	137.239°E	2.2	Sand	5,848	DRR511529
Station 10	37.30511694°N	137.2293169°E	15.3	Mud	4,886	DRR511530
Station 11	37.30498277°N	137.2315828°E	19.6	Sandy mud	9,820	DRR511531
Station 12	37.30469972°N	137.2339669°E	22	Mud	14,489	DRR511532
Station 13	37.30583277°N	137.2373169°E	23.2	Mud	19,671	DRR511533
Station 14	37.30686694°N	137.2355°E	24.3	Sandy mud	38,071	DRR511534
Station 15	37.30786694°N	137.2323°E	17.7	Mud	12,064	DRR511535
Station 16	37.30856694°N	137.2344997°E	15.6	Sandy mud	8,246	DRR511536
Station 17	37.31018277°N	137.2336169°E	7.7	Sandy mud	8,390	DRR511537
Station 18	37.30908277°N	137.2320669°E	17.7	Mud	5,884	DRR511538
Station 19	37.31024999°N	137.2313°E	12.1	Sandy mud	5,721	DRR511539

Proteobacteria, Chloroflexi, and Planctomycetota were the most commonly identified phyla in all the sediment samples, and their total relative abundance accounted for approximately 50% (Fig. 1). Phylum Desulfobacterota, known as sulfate-reducing bacteria, also showed a high abundance (12.2%–18.3%) comparable to the above three phyla in all localities; the average was 14.8%, except for the two stations whose sediment type was sand (Stations 6 and 9). Therefore, it is suggested that the bottom-surface sediments of Tsukumo Bay are relatively reducing environment. The data in this study provide important information about the microbial communities in shallow-sea-reducing sediments.

## Data Availability

The sequences were submitted to the DDBJ Sequence Read Archive (DRA) under the BioProject accession number PRJDB16928 (Table 1).
